# Gradual increasing dyslipidemia in treatment-naive male patients with human immunodeficiency virus and treated with tenofovir plus lamivudine plus efavirenz for 3 years

**DOI:** 10.1186/s13098-021-00756-y

**Published:** 2021-11-18

**Authors:** Dafeng Liu, Xinyi Zhang, Jun Kang, Fengjiao Gao, Yinsheng He, Shenghua He

**Affiliations:** 1 Department of Internal Medicine, The Public and Health Clinic Centre of Chengdu, No. 377 Jingming Road, Jinjiang District, Sichuan 610066 Chengdu, People’s Republic of China; 2 Clinical Medicine, Sichuan University West China Clinical Medical College, Chengdu, China; 3 Department of Infectious Disease, The Public and Health Clinic Centre of Chengdu, No. 377 Jingming Road, Jinjiang District, Sichuan 610066 Chengdu, People’s Republic of China

**Keywords:** Human immunodeficiency virus (HIV), Antiretroviral therapy (ART), Lipid metabolism parameter, Uric acid, Dynamic change, Long-term

## Abstract

**Introduction:**

Since the development of antiretroviral therapy (ART) with TDF plus 3TC plus EFV, this specific regimen has not been studied enough with long-term lipid and uric acid monitoring.

**Methods:**

A prospective follow-up cohort study was performed. Sixty-one treatment-naive male patients with human immunodeficiency virus (HIV) were divided into three groups based on their baseline CD4+ cell count (26, 12, and 23 patients in the < 200, 200 to 350, and > 350 groups, respectively). The lipid and purine metabolism parameters of the patients over 144 weeks were analyzed.

**Result:**

Within 144 weeks, TG, LDL-c, TC and HDL-c gradually increased, especially TC and HDL-c (*P *= 0.001, 0.000, respectively). Moreover, the percentages of hyper-cholesterolemia, hyper LDL cholesterolemia, hyper-triglyceridemia and low HDL cholesterolemia also gradually increased, especially low HDL cholesterolemia significantly increased (*P *= 0.0007). The lower the baseline CD4+ cell counts were, the higher the TG levels and the lower the TC, LDL-c and HDL-c levels were. But there was significant difference of only baseline LDL-c levels between the three groups (*P *= 0.0457). No significant difference of the UA level and the percentages of hyperuricemia was found between the different follow-up time point groups or between the three CD4+ cell counts groups (all *P *> 0.05). The risk factors for dyslipidemia included age, anthropometric parameters and follow-up weeks, and for hyperuricemia was virus load.

**Conclusions:**

Gradual increasing dyslipidemia was found in male patients with human immunodeficiency virus primarily treated with tenofovir plus lamivudine plus efavirenz for 3 years. There-fore lipid metabolism parameters should be closely monitored during long-term ART with the TDF plus 3TC plus EFV regimen.

## Introduction

In recent years, the numbers of patients with human immunodeficiency virus (HIV) and acquired immune deficiency syndrome (AIDS) have sharply increased. By the end of 2019, approximately 38 million people worldwide lived with HIV, and 33 million people died of HIV-related diseases [1]. At the end of October 2019, a total of 1,045,000 patients in China were living with HIV [2, 3]. At the end of September 2018, a total of 262,000 patients died of HIV-related diseases in China [2, 3].

The most effective treatment for AIDS is antiretroviral therapy, which can prolong life expectancy and improve quality of life [4, 5]. When a patient’s CD4+ cell count reaches more than 350/mm^3^ and the viral load reaches undetectable levels within the first year of starting treatment, AIDS patients are predicted to have a normal life expectancy [4, 5]. The cumulative survival rates of AIDS patients have increased markedly [6–8]. As of 2017, 20.9 million patients with HIV had received antiretroviral treatment worldwide. However, metabolic abnormalities, cardiovascular risk factors, and osteoporosis have become important factors affecting the prognosis and quality of life of AIDS patients [9–12].

HIV infection itself and antiretroviral therapy (ART) treatment drugs can cause dyslipidemia. As a first-line ART program launched since the National Twelfth Five-Year Plan in China, the tenofovir (TDF) plus lamivudine (3TC) plus efavirenz (EFV) regimen has a weaker effect on lipid metabolism. Our previous study showed that newly diagnosed male AIDS patients had decreased total cholesterol (TC) levels, uric acid (UA) levels and high-density lipoprotein cholesterol (HDL-c) levels as well as increased triglyceride (TG) levels, especially patients with CD4+ counts < 200/µL. The dyslipidemia and decreased UA levels gradually returned to normal at 4 weeks after initial ART with the TDF plus 3TC plus EFV regimen. Due to the scarcity of female HIV-infected patients who come to the infection clinic of our hospital and have not received antiviral treatment, our previous study lacked the data on the dynamic changes of lipid metabolism and uric acid metabolism in newly diagnosed female AIDS patients after the application of TDF+3TC+EFV. Therefore, this study also did not include female HIV-infected patients. This specific regimen has not been studied enough using long-term lipid and uric monitoring, which is the focus of the current study.

## Patients and methods

### Study population

A prospective cohort study was conducted on sixty-one male patients with HIV who were treatment-naive with the TDF plus 3TC plus EFV regimen at the Public and Health Clinic Centre of Chengdu from October 1, 2012, to December 31, 2017.

The inclusion criteria were as follows: age from 18 to 65 years old; gender is not limited; HIV-1 antibody positive according to enzyme-linked immune-sorbent assay and confirmed by Western blotting; CD4+ T cell count 500 µL within 30 days before enrollment; voluntarily signed informed consent and agreed to undergo follow-up analyses; no plan to move away from current address during the trial; no history of antiretroviral therapy.

The following exclusion criteria were used in this study: patients with acute infections; patients with opportunistic infections or AIDS-related malignant tumors at the time of enrollment; patients with opportunistic infection occurring within 3 months before enrollment and still in unstable condition within 2 weeks before enrollment; patients with hemoglobin <9 g/dL, white blood cell count < 2000/µL, neutrophil count < 1000/µL, platelet count < 75,000/µL, serum creatinine > 1.5-fold upper limit of the normal value (ULN), aspartate aminotransferase/alanine aminotransferase/alkaline phosphatase > threefold ULN, total bilirubin > twofold ULN, serum creatine phosphokinase > twofold ULN, or creatinine clearance rate < 60 mL/min; women who were pregnant or lactating; current drug users; patients with severe mental or neurological diseases; patients with a history of alcoholism; and patients with severe digestive tract ulcers.

AIDS, dyslipidemia and hyperacidemia diagnostic criteria were applied according to the corresponding guidelines [13–15]. According to guidelines, the cutoff values for determining hypercholesterolemia, hyper-low-density lipoprotein cholesterolemia, hypo-high-density lipoprotein cholesterolemia, hypertriglyceridemia and hyperuricemia were as follows: total cholesterol (TC) > 5.18 mmol/L, low-density lipoprotein cholesterol (LDL-c) >  3.37 mmol/L, high-density lipoprotein cholesterol (HDL-c) < 1.0 mmol/L, triglyceride > 1.7 mmol/L, and uric acid (UA) > 420 µmol/L, respectively.

The participants were divided into three groups according to their baseline CD4 T cell counts: there were 26, 12, and 23 patients in the < 200, from 200 to 350 and > 350 cells/µL groups [4, 5, 13], respectively.

### Measurement of anthropometric parameters

The subjects fasted overnight for at least 12 h. At 8:00 a.m. the next day after emptying stool and urine, anthropometric parameters, including height, body weight (BW), body fat weight, lean body mass weight, body mass index (BMI) and body fat percentage, were measured by specially trained researchers using a body fat measuring instrument.

### Detection of laboratory indicators

The subjects fasted overnight for at least 12 h. At 8:00 a.m. the next day, the venous blood of those patients was drawn to measure TC, LDL-c, HDL-c, TG, UA, HIV viral nucleic acid (HIVRNA), and T lymphocyte subsets.

TC, TG, HDL-c, LDL-c and UA levels were measured by the enzymatic method of an automatic biochemical analyzer purchased from Zhejiang Eastern European Biological Products Company. HIVRNA was detected by fluorescent quantitative PCR; T lymphocyte subsets (including CD3+ count, CD4+ count, CD8+ count, CD3+%, CD4+%, CD8+%) were measured by flow cytometry using a Beckman flow cytometer.

The follow-up time points were 0, 4, 8, 12, 24, 36, 48, 60, 72, 84, 96, 108, 120, 132, and 144 weeks after patients underwent ART with the TDF plus 3TC plus EFV regimen [13]. UA levels were measured at each follow-up time point, and TC, LDL-c, HDL-c and TG levels were detected at 0, 24, 48, 96, 120, and 144 weeks [13].

Databases were established according to the needs of the research by two researchers simultaneously collecting and entering data. All of the data were checked by the researchers to assess data integrity, authenticity, and accuracy.

### Patient and public involvement

Patients and the public were involved in the development of the research question or in the design of the study. Patients received oral and written information about this study; however, they were not involved in the recruitment and implementation of the study. In addition, the burden of the intervention was assessed by the investigators. After signing an informed consent form by the participants, they were assessed for eligibility prior to data collection.

### Statistical methods

The Statistical Package for the Social Sciences software version 17.0 (IBM Inc., Armonk, NY, USA) and GraphPad Prism 8 (GraphPad) software were used for statistical analysis. TC, LDL-c, HDL-c, TG and UA levels normally distributed, and statistical analysis was conducted directly. Nonnormally distributed HIVRNA levels were subjected to natural logarithmic transformation before statistical analysis. Quantitative data were expressed as χ ± SD, and categorical data were expressed as rates or percentages. One-way ANOVA was used to compare metabolism parameters from baseline to 144 weeks, and a paired t-test was used to compare metabolism parameters between baseline and some follow-up time points. The Kruskal-Wallis H (K) test for K independent samples was used to compare the percentage of dyslipidemia and hyperuricemia from baseline to 144 weeks. The Mann-Whitney test for two independent samples was used to compare the percentage of dyslipidemia and hyperuricemia between baseline and some follow-up time points. One-way ANOVA was used to compare metabolism parameters between the three different CD4+ T cell count groups at the same time point. Two-way ANOVA was used to compare metabolism parameters between the three different CD4+ T cell count groups from baseline to 144 weeks. A p value < 0.05 was considered statistically significant.

### Ethical consideration

The study was approved by the hospital ethics committee of the Public and Health Clinic Centre of Chengdu (PJ-K2012-012-01). All patients gave written informed consent.

## Results

### Baseline conditions

Sixty-one treatment-naive male patients with HIV in the Public and Health Clinic Centre of Chengdu from October 1, 2012, to December 31, 2017, were divided into three groups according to their baseline CD4+ T cell count: there were 26, 12, and 23 patients in the < 200, 200 to 350, and > 350 cell/µL groups, respectively. There were 42 cases of infection through homosexual contact, 13 cases of infection through heterosexual contact and 5 cases involving both types of sexual contact. The general information, baseline immunity and virological indicators, and lipid metabolism parameters of 61 patients are shown in Table 1.

**Table 1:** Baseline information of male patients with HIV (*n *= 61)

Variable	χ ± SD or cases (%)	Range
Age (years)	32.05 ± 8.38	20–58
Gender (male, %)	61 (100%)	
Infection duration (months)	11.16 ± 1.19	1–86
T lymphocyte subsets		
CD3+ count (cells/µL)	1433.98 ± 595.35	470–3074
CD3+CD4+ count (cells/µL)	313.87 ± 118.473	54–499
CD3+CD4+ percentage (%)	19.78 ± 6.83	1.40–43.40
CD3+CD8+ count (cells/µL)	1119.70 ± 605.0	360–2456
CD3+CD8+ percentage (%)	69.97 ± 13.80	36.13–97.20
Virus load of HIVRNA^a^	41772.77 ± 10.38	895.00–505987.00
Metabolic parameters		
TG (mmol/L)	1.68 ± 1.23	0.39–16.81
TC (mmol/L)	4.20 ± 0.72	2.38–6.09
LDL-c (mmol/L)	2.61 ± 0.68	0.92–4.71
HDL-c (mmol/L)	1.12 ± 0.24	0.57–1.77
UA (µmol/L)	310.72 ± 68.65	143–506
Anthropometric parameters		
Body weight (kg)	62.41 ± 10.53	46–85
Body mass index (kg/m^2^)	21.38 ± 2.82	16.80–28.20
Body fat percentage (%)	15.99 ± 6.32	3–28.4
Body fat weight (kg)	10.40 ± 5.33	1.4–22.10
Body nonfat weight (kg)	51.82 ± 6.67	35.8–64.7
Baseline body mass status		
Body mass index<18 kg/m^2^	3 (4.92)	
24 kg/m^2 ^≥ Body mass index ≥ 18 kg/m^2^	49 (80.33)	
Body mass index > 24 kg/m^2^	9 (14.75)	

TG, triglyceride; TC, total cholesterol; LDL-c, low-density lipoprotein cholesterol; HDL-c, high-density lipoprotein cholesterol; UA, uric acid

^a^Logarithmic transformation before statistical analysis for nonnormally distributed data

### Effectiveness of ART with the TDF plus 3TC plus EFV regimen

**Fig. 1:**
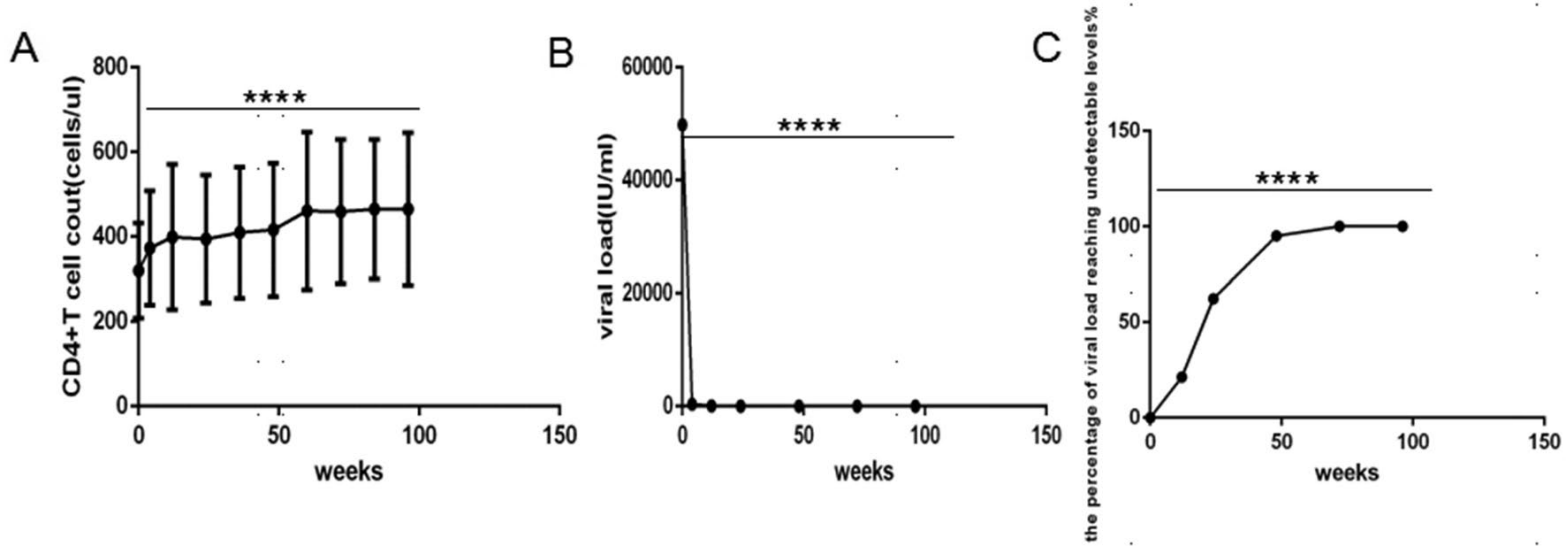
Effectiveness of ART with TDF plus 3TC plus EFV primary treatment for human immunodeficiency virus-infected male patients (*n *= 61). **A** CD4+ T cell count. **B** Viral load. **C** The percentage of viral load reaching undetectable levels. ART, active antiretroviral therapy. TDF, Tenofovir. 3TC, Lamivudine. EFV, Efavirenz. One-way ANOVA was used to compare CD4+ T cell count, viral load and the percentage of viral load reaching undetectable levels between different time points. ****P < 0.0001

In 61 patients, the average CD4+ T cell count (Fig. 1A) gradually increased from 319.80 cell/µL at baseline to 464.85 cell/µL at 96 weeks after ART, and the average viral load (Fig. 1B) decreased rapidly from 49846.32 IU/mL at baseline to undetectable levels (measured by a high-precision detection method) at 72 weeks. The percentage of viral load reaching undetectable levels (Fig. 1C) ranged from 21.31% at 12 weeks to 100.00% at 72 weeks.

### Long-term dynamic changes in anthropometric parameters after treatment with TDF+3TC+EFV

**Fig. 2:**
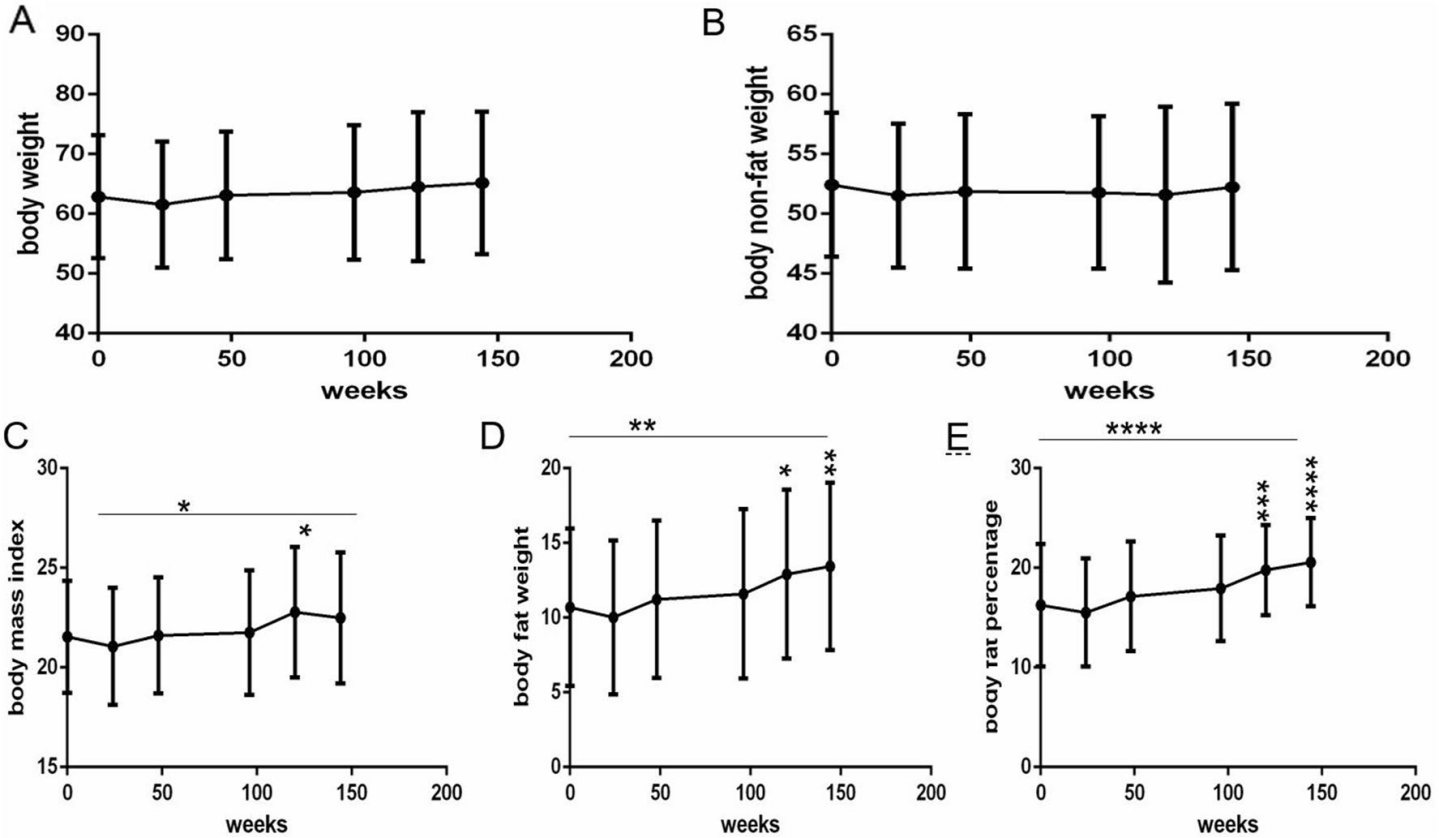
Long-term dynamic changes in anthropometric parameters over 144 weeks after initial ART with TDF plus 3TC plus EFV in treatment-naive patients with HIV (n = 61). **A** Body weight. **B** Body nonfat weight. **C** Body mass index. **D** Body fat weight. **E** Body fat percentage. ART, active antiretroviral therapy. TDF, Tenofovir. 3TC, Lamivudine. EFV, Efavirenz. ANOVA was used to compare anthropometric parameters from baseline to 144 weeks, and a paired t-test was used to compare anthropometric parameters between baseline and some follow-up time points. Comparison between different time points and compared with baseline, * P < 0.05, ** P < 0.01, *** P < 0.001, **** P < 0.0001

The body weight (Fig. 2A) and lean body mass weight (Fig. 2B) of patients did not change significantly over the 144 weeks (all P > 0.05). The body mass index (Fig. 2 C), body fat weight (Fig. 2D) and body fat percentage (Fig. 2E) of patients gradually increased over 144 weeks (all P < 0.05), and the increments were 0.95 kg/m^2^, 2.7 kg, and 4.31%, respectively; body fat weight and body fat percentage, in particular, showed considerable increases.

### Long-term dynamic changes in lipid and purine metabolism parameters after treatment with TDF+3TC+EFV

**Fig. 3:**
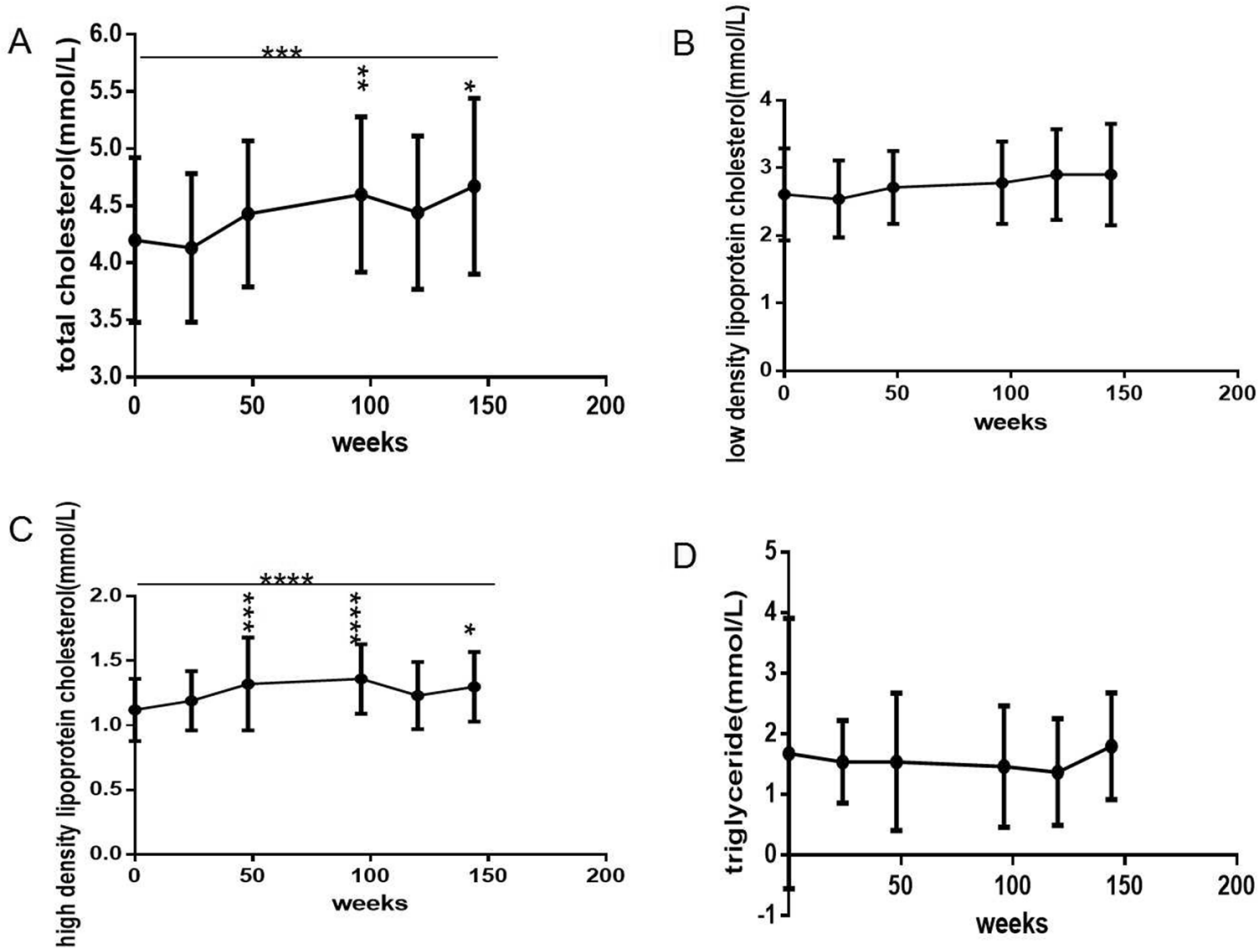
Long-term dynamic changes in lipid metabolic parameters over 144 weeks after initial ART with TDF plus 3TC plus EFV in treatment-naive patients with HIV (*n *= 61). **A** TC level. **B** LDL-c level. **C** HDL-c level. **D** TG level. ART, active antiretroviral therapy. TDF, Tenofovir. 3TC, Lamivudine. EFV, Efavirenz. TC, total cholesterol. LDL-c, low-density lipoprotein cholesterol. HDL-c, high-density lipoprotein cholesterol. TG, triglyceride. ANOVA was used to compare lipid metabolism parameters from baseline to 144 weeks, and a paired t-test was used to compare lipid metabolism parameters between baseline and some follow-up time points. Comparison between different time points and compared with baseline, *P < 0.05, ** P < 0.01, ***P < 0.001, ****P < 0.0001

**Fig. 5:**
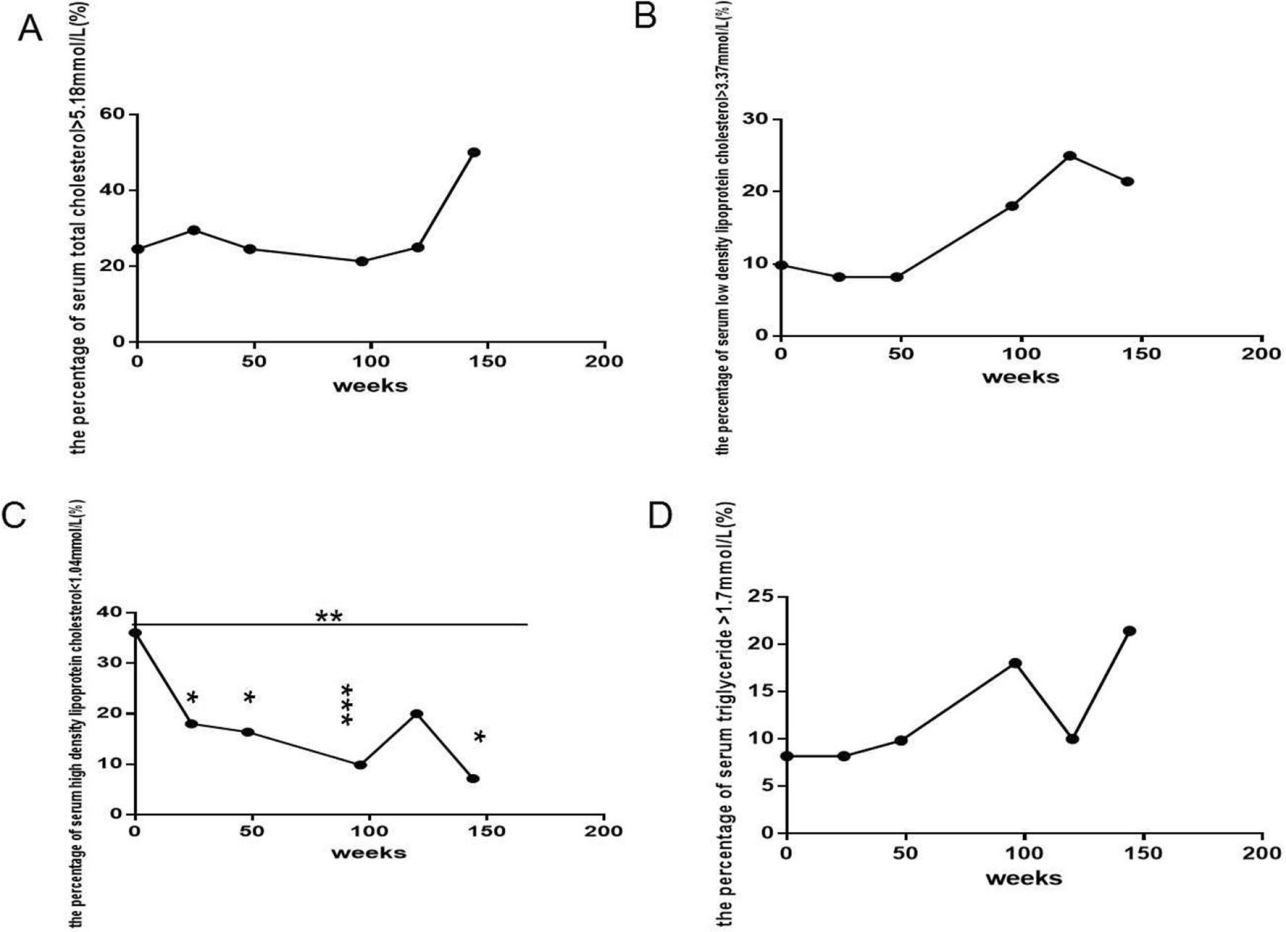
Long-term dynamic changes in UA and the percentage of hyperuricemia (UA ˃ 420 µmol/L) over 144 weeks after initial ART with TDF plus 3TC plus EFV in treatment-naive patients with HIV (*n *= 61). **A** UA level. **B** the percentage of hyperuricemia. ART, active antiretroviral therapy. TDF, Tenofovir. 3TC, Lamivudine. EFV, Efavirenz. UA, uric acid. ANOVA was used to compare UA levels from baseline to 144 weeks, and a paired t-test was used to compare UA levels between baseline and some follow-up time points. The Kruskal-Wallis H(K) test for K independent samples was used to compare the percentage of hyperuricemia from baseline to 144 weeks. The Mann-Whitney test for two independent samples was used to compare the percentage of hyperuricemia between baseline and follow-up time points. Comparison between different time points and compared with baseline, all P > 0.05

TC, LDL-c, HDL-c, TG and UA levels (Figs. 3A–D and 5A) all gradually increased with prolonged ART, but the increases were small, and only the increases in TC levels (Fig. 3A) and HDL-c levels (Fig. 3C) were statistically significant (*P *= 0.0007, 0.000, respectively). Compared with baseline, there were significant differences in TC levels at 96 and 144 weeks (Fig. 3A) and in HDL-c levels at 48, 96 and 144 weeks (Fig. 3C) (*P *= 0.002, 0.0329, 0.0004, 0.0001, 0.0157, respectively). There were no significant differences in LDL-c (Fig. 3B), TG (Fig. 3D) or UA levels (Fig. 5A) between different time points compared with baseline (all *P *> 0.05).

**Fig. 4:**
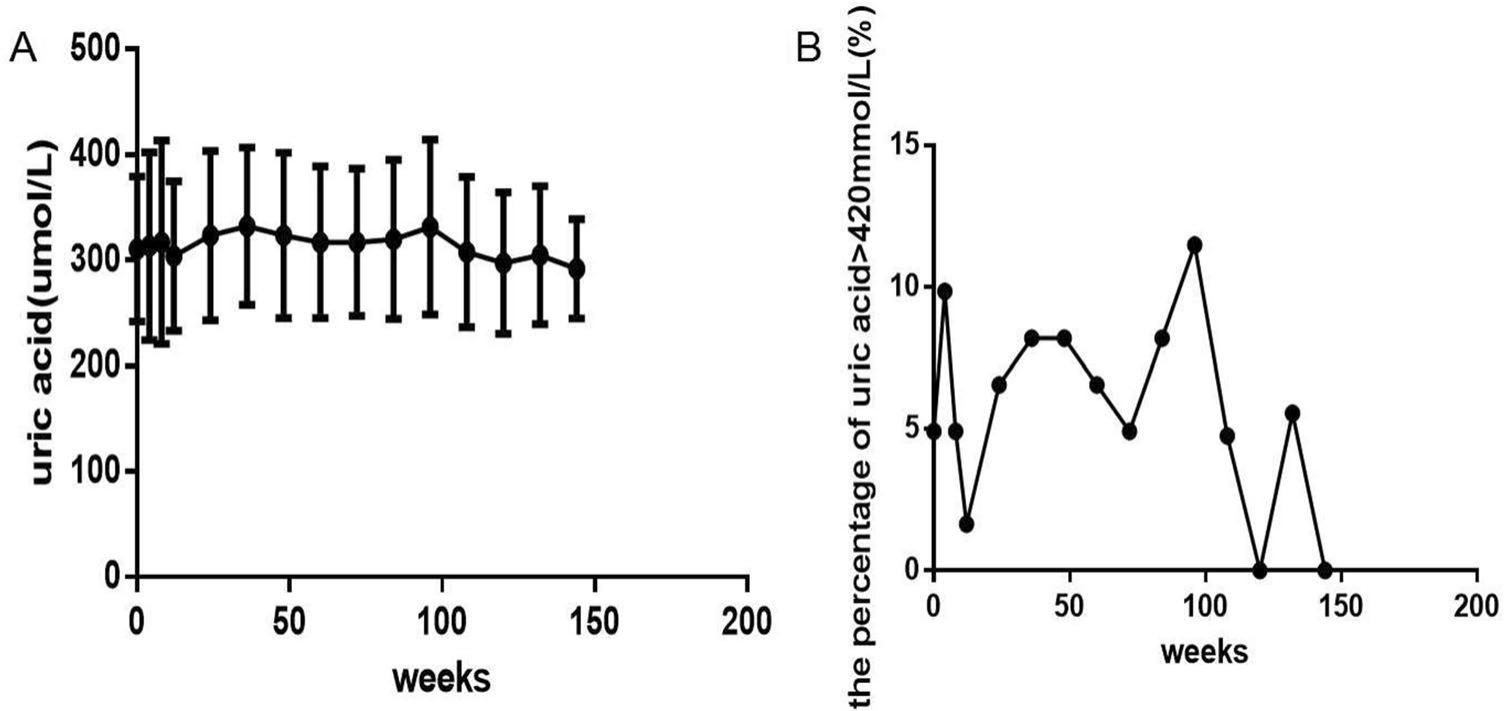
Long-term dynamic changes in the percentage of dyslipidemia over 144 weeks after initial ART with TDF plus 3TC plus EFV in treatment-naive patients with HIV (*n *= 61). **A** The percentage of hypercholesterolemia (TC > 5.18 mmol/L). **B** The percentage of hyper low-density lipoprotein cholesterolemia (LDL-c > 3.37 mmol/L). **C** The percentage of hypo-high-density lipoprotein cholesterolemia (HDL-c < 1.00 mmol/L). **D** the percentage of hypertriglyceridemia (TG > 1.7 mmol/L). ART, active antiretroviral therapy. TDF, Tenofovir. 3TC, Lamivudine. EFV, Efavirenz. TC, total cholesterol. LDL-c, low-density lipoprotein cholesterol. HDL-c, high-density lipoprotein cholesterol. TG, triglyceride. The Kruskal-Wallis H(K) test for K independent samples was used to compare the percentage of dyslipidemia from baseline to 144 weeks. The Mann-Whitney test for two independent samples was used to compare the percentage of dyslipidemia between baseline and follow-up time points. Comparison between different time points and compared with baseline, *P < 0.05, ** P < 0.01, ***P < 0.001

The percentages of hypercholesterolemia (Fig. 4A), hyper LDL cholesterolemia (Fig. 4B) and hypertriglyceridemia (Fig. 4D) all gradually increased, but the increases were not significant (all *P *> 0.05). In contrast, the percentage of hypo-HDL cholesterolemia (Fig. 4C) gradually decreased with prolonged ART, and a significant difference was found from baseline to 144 weeks (Fig. 4C) (*P *= 0.0007) and at 24, 48, 96, 144 weeks compared with baseline (Fig. 4C) (*P *= 0.026, 0.014, 0.001, 0.036, respectively). The percentage of hyperuricemia (Fig. 5B) slightly decreased with the extension of the ART treatment time, but this decrease was not significant (*P *> 0.05).

### Effect of baseline CD4+ cell count on lipid and purine metabolic parameters after treatment with TDF+ 3TC+EFV

**Fig. 6:**
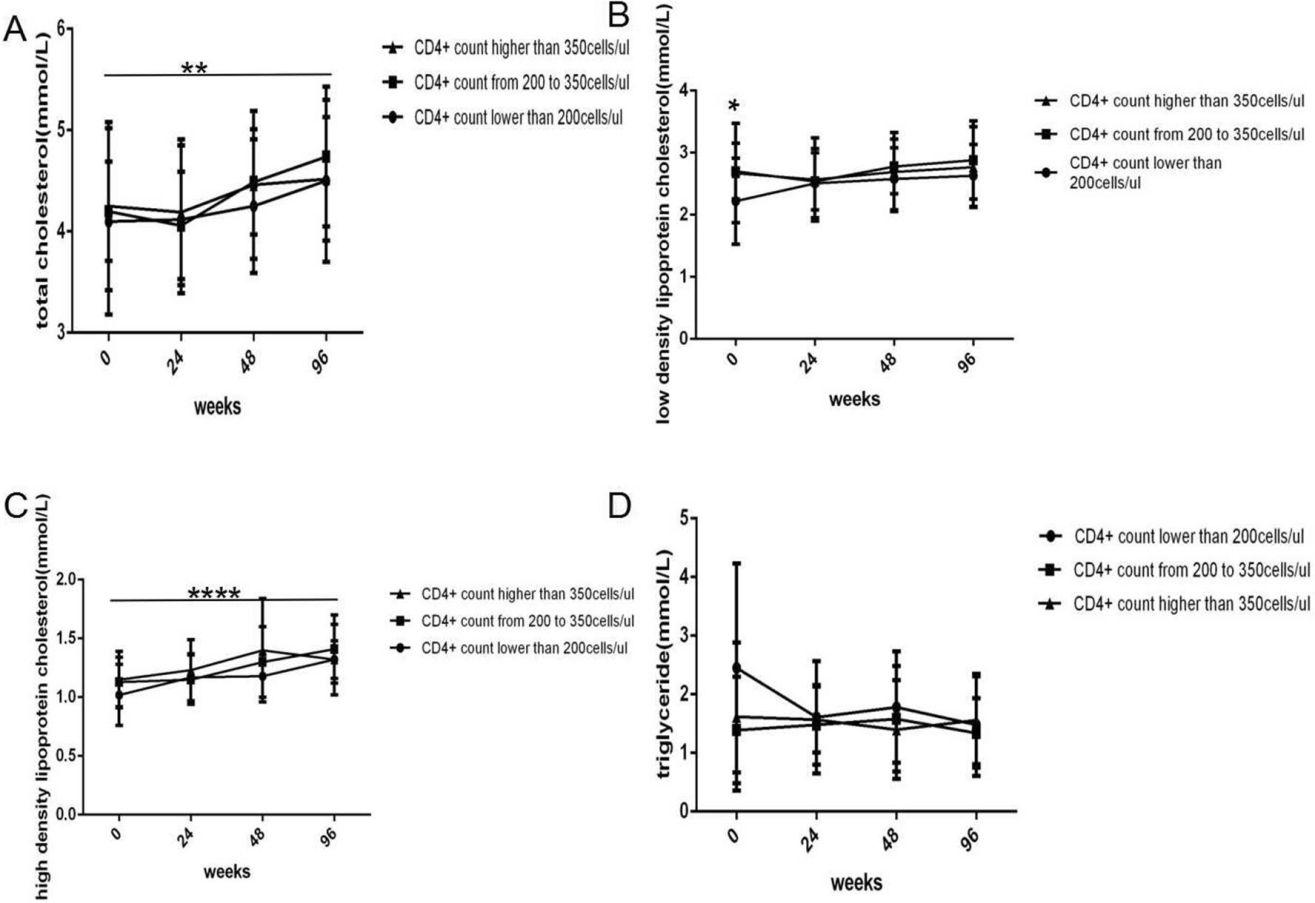
Long-term effect of baseline CD4+ T cell count on lipid metabolism parameters within 96 weeks after initial ART with TDF plus 3TC plus EFV in treatment-naive patients with HIV (*n *= 61; 26, 12, 23 cases in the <  200, 200 to 350, and > 350 groups, respectively). **A** TC level. **B** LDL-c level. **C** HDL-c level. **D** TG level. ART, active antiretroviral therapy. TDF, Tenofovir. 3TC, Lamivudine. EFV, Efavirenz. TC, total cholesterol. LDL-c, low-density lipoprotein cholesterol. HDL-c, high-density lipoprotein cholesterol. TG, triglyceride. One-way ANOVA was used to compare lipid metabolism parameters between three groups at the same time point, and Two-way ANOVA was used to compare lipid metabolism parameters between three groups from baseline to 96 weeks. Comparison between different time points and different CD4+ T cell count groups, *P < 0.05, ** P< 0.01, ****P < 0.0001

**Fig. 7:**
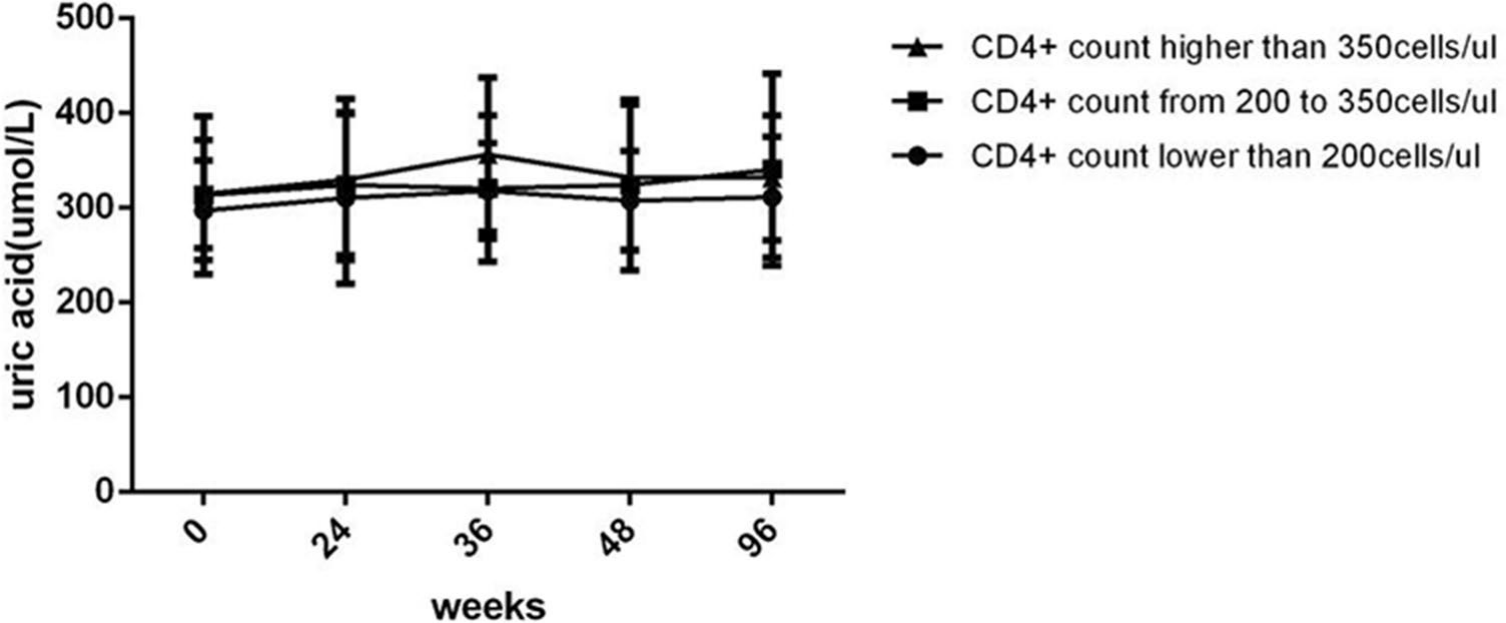
Long-term effect of baseline CD4+ T cell count on UA level within 96 weeks after initial ART with TDF plus 3TC plus EFV in treatment-naive patients with HIV (*n *= 61; 26, 12, 23 cases in < 200, from 200 to 350, > 350 groups, respectively). Abbreviations: ART, active antiretroviral therapy. TDF, Tenofovir. 3TC, Lamivudine. EFV, Efavirenz. UA, uric acid. One-way ANOVA was used to compare UA between three groups at the same time point, and Two-way ANOVA was used to compare UA between three groups from baseline to 96 weeks. Comparison between different time points and different CD4+ T cell count groups, all P > 0.05

The lower the CD4+ cell count at baseline was, the higher the TG levels (Fig. 6D) and the lower the TC (Fig. 6A), LDL-c (Fig. 6B), HDL-c (Fig. 6C) and UA levels (Fig. 7) were; moreover, these changes were maintained throughout the follow-up period after ART treatment. However, there was no significant difference in the change from baseline to 96 weeks between the three different CD4+ cell count groups (all *P *> 0.05). The difference of the LDL-c levels (Fig. 6B) at baseline was significant between the three different CD4+ cell count groups (*P *= 0.0457). TC levels (Fig. 6A) and HDL-c levels (Fig. 6C) all gradually increased along with prolonged ART regardless of the CD4+ cell count at baseline.

### The risk factors of lipid and purine metabolic parameters

According to Spearman correlation analysis, nonalcoholic fatty liver disease, age, body weight, BMI, body fat weight, and body fat percentage were all positively correlated with TC, TG and LDL-c levels; lean body mass weight was positively correlated with LDL-c levels; and follow-up duration was positively correlated with TC and HDL-c levels. In contrast, nonalcoholic fatty liver disease, body weight, BMI, body fat weight, body fat percentage and lean body mass weight were all negatively correlated with HDL-c levels (Table 2). Based on multiple stepwise regression analysis, the risk factors for TC levels included body weight, age, lean body mass weight and follow-up duration, for TG levels included body weight, lean body mass weight, BMI and age, for HDL-c levels included BMI, CD3+CD4+ count and body weight, and for LDL-c levels included BMI and follow-up duration (Table 3).

**Table 2:** Spearman correlation analysis between lipid metabolism parameters and age, anthropometric parameters, and immunological and virological indicators (*n *= 61)

Variable	TC (mmol/L)		TG (mmol/L)		HDL-c (mmol/L)		LDL-c (mmol/L)		UA (µmol/L)	
	r	p	r	p	r	p	r	p	r	p
NAFLD (1 = without, 2 = with)	0.357	0.002	0.353	0.003	− 0.255	0.033	0.251	0.038	0.320	<0.0001
Age (year)	0.304	< 0.0001	0.164	0.004			0.226	<0.0001	− 0.151	<0.0001
Body weight (kg)	0.243	0.013	0.241	0.014	− 0.413	<0.0001	0.341	<0.0001	0.338	<0.0001
Body mass index (kg/m^2^)	0.343	0.001	0.236	0.021			0.418	<0.0001		
Body fat percentage (%)	0.318	0.002	0.460	<0.0001	− 0.238	0.020	0.283	0.006		
Body fat weight (kg)	0.312	0.002	0.390	<0.0001	− 0.338	0.001	0.326	0.001		
Body nonfat weight (kg)					− 0.378	<0.0001	0.319	0.002	0.377	<0.0001
CD3+CD4+ count (cells/µl)									0.161	<0.0001
Virus load (IU/ml)									0.306	0.001
Follow-up duration	0.192	<0.0001			0.281	<0.0001				

NAFLD, nonalcoholic fatty liver disease. TG, triglyceride. TC, total cholesterol. LDL-c, low-density lipoprotein cholesterol. HDL-c, high-density lipoprotein cholesterol. UA, uric acid

**Table 3:** Multiple stepwise regression analysis of influencing factors, including age, anthropometric parameters, immunological and virological indicators on lipid and uric metabolism parameters (n = 61)

Independent variable		B	Std. error	Beta	t	p
TC (mmol/L)	Constant	3.224	0.681	–	4.733	<0.0001
	Body weight (kg)	0.053	0.017	0.781	3.161	0.002
	Age (year)	0.031	0.007	0.369	4.249	<0.0001
	Body nonfat weight (kg)	− 0.064	0.028	− 0.578	− 2.319	0.023
	Follow-up weeks	0.006	0.002	0.269	3.308	0.003
TG (mmol/L)	Constant	18.767	5.130	–	3.658	0.001
	Body weight (kg)	0.902	0.192	3.564	4.696	<0.0001
	Body nonfat weight (kg)	− 0.768	0.157	− 1.883	− 4.889	<0.0001
	Body mass index (kg/m^2^)	− 1.684	0.477	− 1.798	− 3.528	0.001
	Age (year)	0.095	0.042	0.293	2.249	0.031
HDL-c (mmol/L)	Constant	1.153	0.169	–	6.805	<0.0001
	Body mass index (kg/m^2^)	0.070	0.015	0.791	4.699	<0.0001
	CD3+CD4+ count (cells/µL)	0.001	0.000	0.423	4.930	<0.0001
	Body weight (kg)	− 0.028	0.004	− 1.145	− 6.174	<0.0001
LDL-c (mmol/L)	Constant	1.056	0.374	–	2.827	0.006
	Body mass index (kg/m^2^)	0.073	0.017	0.396	4.222	<0.0001
	Follow-up weeks	0.003	0.002	0.173	1.847	0.068
UA (µmol/L)	Constant	323.772	15.315	–	21.141	<0.0001
	Virus load (IU/mL)	0.000	0.000	0.313	2.032	0.049

TG, triglyceride; TC, total cholesterol; LDL-c, low-density lipoprotein cholesterol; HDL-c, high-density lipoprotein cholesterol; UA, uric acid

In addition, nonalcoholic fatty liver disease, body weight, lean body mass weight, CD3+CD4+ cell count and virus load were all positively correlated with UA levels, while age was negatively correlated with UA levels (Table 2). Based on multiple stepwise regression analysis, only virus load was a risk factor for UA (Table 3).

## Discussion

Currently, most of the literature focuses on the proportion dyslipidemia in patients treated with ART. As reported in the literature, the incidence of dyslipidemia in HIV/AIDS patients is quite high in Asia, especially in some southeast Asian countries, such Thailand (34.93%) [16], Tanzania (more than 76%) [17], southern Ethiopia (82.3%) [18] and India (20-100%) [19–22]. It has also been reported that 10–60% of patients receiving ART treatment have hypercholesterolemia [8, 23–26], 20-70% have hypertriglyceridemia [8, 9, 26, 27], 35.1% have hyper-low-density lipoprotein cholesterolemia [26], and 20-68.5% have hypo-high-density lipoprotein cholesterolemia [9, 26–29]. Although the patients in the above literature included male and female participants, male participants accounted for the vast majority. In some literature, more than 93% of the participants were male. Therefore, we think it is reasonable to discuss research conducted on male participants and research conducted on male and female participants. No study has used long-term lipid and uric monitoring with specific first-line ART regimens, especially the TDF+3TC+EFV regimen.

In this prospective 3-year follow-up cohort study was the first to report the use of long-term lipid and uric monitoring to assess the efficacy of the TDF+3TC+EFV regimen. The results showed that in patients treated with the TDF+3TC+EFV regimen for 3 years, TC, LDL-c and HDL-c levels gradually increased, especially TC and HDL-c levels. TG levels first gradually decreased and then gradually increased. Moreover, the percentages of hypercholesterolemia, hyper-LDL cholesterolemia and hypertriglyceridemia all gradually increased, while the percentage of hypo-HDL cholesterolemia gradually decreased. This shows that in the early stage of ART treatment, disordered lipid metabolism was improved, especially hypo-HDL cholesterolemia. However, along with prolonged ART, the proportion of hyperlipidemia and hypertriglyceridemia gradually increased, especially hyper-LDL cholesterolemia and hypertriglyceridemia after 48 weeks and hypercholesterolemia after 120 weeks, which ranged from 9.84%, 8.2%, and 24.59% at baseline to 21.43%, 21.43%, and 50.00% at 144 weeks, respectively.

This alternate cause of early lipid metabolism may be partially due to the changes in not weight gain but appetite improvement among patients who contracted AIDS after ART treatment. Because within 48 weeks after ART, all anthropometric parameters, including body weight, body fat weight, lean body mass weight, body fat percentage and BMI, did not increase. While the long-term characteristics of lipid and purine metabolism may be partially due to the changes in weight gain among patients who contracted AIDS after ART treatment. Because among those patients body weight increased by 2.81 kg, and body fat weight increased by 2.74 kg from 48 to 144 weeks, but lean body mass weight did not increase over the 144 weeks.

This finding was consistent with the literature report that among patients living with HIV for a mean duration of 17.4 years, 35.6% had ASCVD, and of those without ASCVD, 53–86% had intermediate or moderate-to-high 10-year ASCVD risk scores, cardiovascular risk factors including HIV, 31.9% had low high-density lipoprotein cholesterol levels, and 79.3% needed to receive statin therapy [30].

In this prospective 3-year follow-up cohort study we assessed baseline CD4+ cell count, age, anthropometric parameters, and immunological and virological indicators impacting on lipid and purine metabolic parameters in male patients with HIV undergoing primary treatment, and found that the lower the baseline CD4+ cell count was, the higher the TG levels were and the lower the TC, LDL-c and HDL-c levels were. The risk factors for lipid and purine metabolic parameters included age, anthropometric parameters, and immunological and virological indicators. After comparisons between different time points over the 144 weeks and comparisons with baseline levels, no significant difference in UA levels was found. The lower the baseline CD4+ cell count was, the higher the UA levels were, and these changes were maintained throughout the follow-up period after ART treatment; however, there was no statistical significance in the change from baseline to 144 weeks between the three different CD4+ T cell count groups. That is, the TDF+3TC+EFV regimen and CD4+ T cell count at baseline had no long-term dynamic effects on purine metabolism. This finding was inconsistent with the literature report that a high CD4+ cell count was a risk factor for hypertriglyceridemia, while a CD4+ cell count less than 200 copies/mm^3^ increased the risk of hypercholesterolemia [26]. Regardless of whether the initial treatment regimen was based on D4T, the risk of hyperlipidemia in HIV/AIDS patients aged 50 and above was significantly higher than that in young HIV/AIDS patients aged under 40 years [31].

To our knowledge, this prospective follow-up cohort study is the first to investigate this specific regimen with TDF plus 3TC plus EFV using with long-term lipid and uric acid monitoring and the risk factors for lipid and uric acid. The results showed that gradual increasing dyslipidemia was found in male patients with human immunodeficiency virus primarily treated with tenofovir plus lamivudine plus efavirenz for 3 years. The risk factors for dyslipidemia included age, anthropometric parameters and follow-up weeks, and for hyperuricemia was virus load. There-fore lipid metabolism parameters should be closely monitored during long-term ART with the TDF plus 3TC plus EFV regimen.

In this cohort study the proportion of patients with abnormal lipid metabolism during the whole study period was less than that reported in the literature. The reasons may be related to the younger age of patients in this cohort, the duration of follow-up was not too long, and the TDF+3TC+EFV regimen had little effect on lipid metabolism.

The present study had some limitations. The sample size was small, and this was a single-center cohort study that only involved the TDF+3TC+EFV regimen and male patients, and the duration of follow-up was not too long. Further multicenter studies, more ART regimens, increase the number of female patients and large-sample randomized controlled trials and longer the duration of follow-up are necessary.

## Conclusions

Gradual increasing dyslipidemia was found in male patients with human immunodeficiency virus primarily treated with tenofovir plus lamivudine plus efavirenz for 3 years. The risk factors for dyslipidemia included age, anthropometric parameters and follow-up weeks, and for hyperuricemia was virus load. Therefore lipid metabolism parameters should be closely monitored during long-term ART with the TDF plus 3TC plus EFV regimen.

## Data Availability

All data, models, or code generated or used during the study are available from the corresponding author by request: Dafeng Liu, E-mail: liudf312@126.com.

## Abbreviations

AIDS: Acquired immunodeficiency syndrome
ART: Antiretroviral therapy
BMI: Body mass index
BW: Body weight
EFV: Efavirenz
HDL-c: High-density lipoprotein cholesterol
HIV: Human immunodeficiency virus
HIVRNA: HIV viral nucleic acid
3TC: Lamivudine
LDL-c: Low-density lipoprotein cholesterol
TC: Total cholesterol
TDF: Tenofovir
TG: Triglycerides
ULN: Normal value
UA: Uric acid
